# Comparative Ionomics and Metabolic Responses and Adaptive Strategies of Cotton to Salt and Alkali Stress

**DOI:** 10.3389/fpls.2022.871387

**Published:** 2022-04-25

**Authors:** Jiaxin Guo, Xiaoyu Lu, Yifan Tao, Huijuan Guo, Wei Min

**Affiliations:** Department of Resources and Environmental Science, Shihezi University, Shihezi, China

**Keywords:** salt-alkali stress, cotton, ionome, metabolome, organic acid

## Abstract

Soil salinization and alkalization severely inhibit agriculture. However, the response mechanisms of cotton to salt stress or alkali stress are unclear. Ionomics and metabolomics were used to investigate salt and alkali stresses in cotton roots and leaves. Compared with the control, salt-treated and alkali-treated cotton plants showed 51.8 and 53.0% decreases in biomass, respectively. Under salt stress, the concentration of N decreased in roots but increased in leaves, and the concentrations of P and K increased in roots but decreased in leaves. Salt stress inhibited Ca, B, N, and Fe uptake and Mg, K, P, S, and Cu transport, but promoted Mo, Mn, Zn, Mg, K, P, S, and Cu uptake and Mo, Mn, Zn, B, N, and Fe transport. Under alkali stress, the concentrations of N and P in roots and leaves decreased, while the concentrations of K in roots and leaves increased. Alkali stress inhibited P, Ca, S, N, Fe, and Zn uptake and N, P, Mg and B transport, but promoted K, Mn, Cu, Mo, Mg, and B uptake and K, Mn, Cu, Mo, Fe, and Zn transport. Under salt stress in the leaves, 93 metabolites increased, mainly organic acids, amino acids, and sugars, increased in abundance, while 6 decreased. In the roots, 72 metabolites increased, mainly amino acids, organic acids, and sugars, while 18 decreased. Under alkali stress, in the leaves, 96 metabolites increased, including organic acids, amino acids, and sugars, 83 metabolites decreased, including organic acids, amino acids, and sugars; In the roots, 108 metabolites increased, including organic acids, amino acids, and sugars. 83 metabolites decreased, including organic acids and amino acids. Under salt stress, cotton adapts to osmotic stress through the accumulation of organic acids, amino acids and sugars, while under alkali stress, osmoregulation was achieved *via* inorganic ion accumulation. Under salt stress, significant metabolic pathways in the leaves and roots were associated with amino acid and organic acid metabolism, sugar metabolism was mainly used as a source of energy, while under alkali stress, the pathways in the leaves were related to amino acid and linoleic acid metabolism, β-Oxidation, TCA cycle, and glycolysis were enhanced to provide the energy needed for life activities. Enhancing organic acid accumulation and metabolism in the roots is the key response mechanism of cotton to alkalinity.

## Introduction

High salinity and alkalinity cause abiotic stresses that seriously affect agricultural production (Rolly et al., 2020). It is estimated that 1.13 × 10^9^ ha of the world’s territory is influenced by salt or alkali stress, representing > 20% of the total arable land (Morton et al., 2019). The total area of salt–alkali land in China is ∼9.91 × 10^7^ ha (Yang et al., 2021), and Xinjiang Province is greatly affected by soil salinization and alkalization, where the salt–alkali land in irrigated areas accounts for 37.72% of the total arable land (Yang et al., 2016). Cotton (*Gossypium hirsutum* L.) is one of the most important cash crops in Xinjiang, the cultivation area of which accounts for > 82.8% of the national total. Although cotton plants are salt-tolerant, their growth and development can be severely affected by salt and alkali stresses (Ashraf et al., 2018). Soil salinization and alkalization frequently co-occur, and it is generally believed that salt stress is caused by neutral salts (NaCl and Na_2_SO_4_), whereas alkali stress is induced by alkali salts (NaHCO_3_ and Na_2_CO_3_) (Yang et al., 2007; Lin et al., 2016). Compared with salt stress, alkali stress causes greater damage to plants (Wang et al., 2017). Therefore, investigating the differential effects of salt and alkali stresses on cotton is important for guiding agricultural production.

Salt stress causes osmotic stress and ion toxicity by disrupting ion homeostasis in plant cells (Ruiz et al., 2016). Plants attempt to re-establish osmotic and ionic homeostasis to adapt to the new environment by accumulating small-molecule soluble substances such as betaine, proline, polyamines, and polyols for osmoregulation (Lacerda et al., 2003; Shen et al., 2018). Meanwhile, cells rapidly accumulate inorganic ions such as Na^+^, K^+^, and Ca^2+^ (Shabala and Shabala, 2011; Chen et al., 2017). However, excessive Na^+^ and Cl^–^ can disrupt the ion homeostasis in plants (Ruiz et al., 2016) and interfere with the influx or metabolism of other essential ions (Zhao et al., 2020). Studies on ion homeostasis under salt or alkali stress have focused on the mechanisms of Na^+^ and K^+^ transport in plants (Zhao et al., 2014). However, the maintenance of intracellular ion homeostasis involves a whole range of ions, not just one or several ions. Ionomics represents an important approach for understanding ion homeostasis in plants (Wu et al., 2013a). Under salt stress, the concentration of Na in cotton roots, stems, and leaves increases significantly, while the concentrations of K, Cu, B, and Mo in the roots and Mg and S in the leaves decrease drastically (Guo et al., 2019). Some stress factors are shared by alkali and salt stresses. However, the high pH associated with alkali stress causes the precipitation of many nutrient ions (Ca^2+^, Mg^2+^, Fe^2+^, Mn^2+^, Cu^2+^, and Zn^2+^) in the rhizosphere, causing nutrient deprivation and further damage to crops (Wang et al., 2012; Liu et al., 2019; Guo et al., 2020). There are few studies on the differences in osmoregulation in cotton under salt and alkali stresses. The uptake and transport of ions and the relative osmoregulatory capacity of ions and small-molecule soluble substances have not been clarified.

Metabolomics describes the changes in metabolites and metabolic pathways *in vivo* and reveals the connections between organisms and the environment. Plants increase the activity of certain metabolic pathways to adapt to salt stress, such as amino acid metabolism, tricarboxylic acid (TCA) cycle, glycolysis, signal transduction, and sugar metabolism (Kumari et al., 2015). In the leaves, sugar metabolism, photosynthesis, and TCA cycle related to energy supply are inhibited under salt stress (Pang et al., 2016). Under alkali stress, β-oxidation, glycolysis, citric acid cycle, and amino acid metabolism serve important roles in improving alkali tolerance (Zhang et al., 2016; Xiao et al., 2020). Enhanced glycolysis process provided more carbon source and energy for alkali stress response of roots (Lu et al., 2021). Meanwhile, Sucrose acted as a signaling molecule and directly mediated amino acid metabolism (Jia et al., 2019), and other small-molecule soluble substances such as organic acids, amino acids, betaine, sugars, and alcohols are synthesized and accumulated (Munns and Tester, 2008; Sun et al., 2019). Most studies suggest that the enhanced accumulation and metabolism of organic acids in the roots contribute to intracellular ion homeostasis and counteract the negative effects of high pH under alkali stress, which is an important mechanism for adaptation to alkali conditions (Guo et al., 2018; Han et al., 2019; Xiang et al., 2019). However, the effect of salt or alkali stress on amino acid metabolism in plant roots remains inconclusive. Guo et al. (2017) reported that alkali stress inhibited nitrogen metabolism and amino acid production in the roots, while Wang et al. (2021) showed that amino acid and organic acid metabolic pathways were critical for the roots under alkali stress. These studies suggest that the changes of other metabolites in plants and the relationship between their changes and the secretion of organic acids by roots are still unclear, and plants coordinate all organs and most metabolic processes to adapt to salt and alkali stresses, and the transformation of metabolites within plant organs needs to be further investigated.

Although salt and alkali tolerance in cotton have been intensively studied, the differences between the mechanisms of such tolerance have not been fully elucidated, and few studies have evaluated the changes in the roots and leaves under salt and alkali stresses. Therefore, the purpose of this study was to investigate: (i) the effects of salt and alkali stresses on ion uptake, transport, and allocation in cotton; (ii) the effects of salt and alkali stresses on metabolic pathways and metabolites in cotton leaves and roots; and (iii) the conversion between metabolites and the links between metabolic pathways in the leaves and roots. By evaluating ion uptake and transport as well as the differential metabolites and metabolic pathways, this study provides insights into the mechanisms and regulatory pathways of the response of cotton to salt and alkali stresses.

## Materials and Methods

### Experimental Site and Soil Description

A soil column experiment was conducted in 2020 in a greenhouse at the experimental station of the Agricultural College of Shihezi University (44°18′41″N, 86°3′18″E). The climate is temperate arid zone with a mean annual temperature of 7.8°C, precipitation of 210 mm, and evaporation of 1,660 mm, with little annual variation. During the experiment, the maximum temperature and minimum temperature of the greenhouse were 15.8 and 35.6°C, respectively. The sample soil collected from the experimental station was calcaric fluvisol with a loam texture. The basic physicochemical properties of the soil were as follows: organic matter, 14.9 g⋅kg^–1^; total nitrogen, 1.1 g⋅kg^–1^; available phosphorus, 10.6 mg⋅kg^–1^; and available potassium, 244 mg⋅kg^–1^. The experimental plant was the cotton cultivar “Lumianyan24.”

### Experimental Design

Three treatments were used in this experiment: (1) control (CK, untreated soil); (2) salt soil (CS, soil + NaCl); and (3) alkali soil (AS, soil + Na_2_CO_3_ + NaHCO_3_). Each treatment was in triplicate.

The soil was naturally air-dried, ground, and passed through a 2-mm sieve before experimentation. NaCl or Na_2_CO_3_ + NaHCO_3_ (weight ratio, 1:1) solution was added to the soil until saturation (the same volume of deionized water was added to the control). The soil was left for 1 month to reach equilibrium and then used as the salt or alkali soil. The soil was then air-dried, ground, and sieved. Soil samples were collected to measure salinity, conductivity, and pH. The specific experimental treatments are shown in Table 1.

**TABLE 1:** Type and degree of salinity and alkalinity in the soil under different treatments.

Treatment	Salinity and alkalinity	Salt content (g kg ^– 1^)	EC_1:5_ (dS m ^– 1^)	pH (1:2.5)
CK	Control: normal soil	0.53	0.17	8.16
CS	NaCl: moderate salinization	4.43	1.39	8.43
AS	Na_2_CO_3_+NaHCO_3_: moderate alkalization	2.03	0.63	9.92

*CK, control treatment without salt or alkali stress; CS, NaCl stress treatment; AS, Na_2_CO_3_ + NaHCO_3_ stress treatment.*

A simulation of a cotton plantation was performed in a soil column (a cylindrical container with a height of 60 cm, a diameter of 35 cm, and a closed bottom). Each soil column was packed with 60 kg of air-dried soil in 10-cm layers (a total depth of 50 cm) at a density of 1.25 g⋅cm^–3^. Drip irrigation was performed using a horizontal capillary tube placed above the soil column, with an emitter fixed at the center of the column. Each column was irrigated with one emitter at 2.5 L per column. On April 10, 2020, 20 seeds were sown in dry soil in each column and irrigated for seed germination. When the cotton seedlings reached the “two leaves and one heart” stage, two cotton seedlings with uniform growth were retained in each column. To ensure adequate water supply, drip irrigation was used regularly for watering during the experiment to keep the soil moisture at 60–80% of field capacity. The experiment was terminated at 60 d after sowing.

### Plant Materials

Samples were collected at the seedling stage and began at 10:30 in the CK treatment with collection of samples from, the CS treatment beginning at approximately 12:30, the AS treatment beginning at approximately 12:30 and concluding at approximately 14:30. Three representative cotton plants were collected from each treatment and divided into three parts: roots, stems, and leaves. While in the field, collected samples were stored on ice in coolers. Some leaf samples were freeze-dried in liquid nitrogen, ground into powder using a plant grinder (Scientz-48, Tongyuan Juwu Technology, Beijing, China), and stored at −80°C for later use.

### Determination Method

#### Measurement of Biomass

To determine the dry matter of cotton, The roots, stems, and leaves was washed with distilled water and then dried in an oven at 70 °C for 48 h, weighed.

#### Measurement of Ions Content

Plant samples were digested with concentrated H_2_SO_4_-H_2_O_2_, and the N content was determined using a Hanon K9840 semi-auto Kjeldahl analyzer (Jinan Hanon Instruments Co., Ltd., China). For the measurement of Na, P, K, Ca, Mg, S, Fe, Mn, Zn, Cu, B, and Mo, 100 mg of cotton plant sample was combined with 1.5 mL concentrated nitric acid and heated on an electric hotplate at 145°C to remove part of the organic matter, and then evaporated to dryness. The sample was then combined with 1 mL concentrated nitric acid and sealed in a digestion tank, which was placed in an oven at 195°C for 12 h of digestion, and the sample was evaporated to dryness. The sample was completely dissolved in 0.5 mL concentrated nitric acid plus 0.5 mL internal standard and 3 mL deionized water, 2 mL of which was diluted to 10 mL with 18 mΩ ultrapure water. Finally, the metal content was determined using inductively coupled plasma-mass spectrometry (Agilent 7900 ICP-MS, United States) according to the manufacturer’s instructions.

#### Extraction and Measurement of Metabolites

For metabolite extraction, 50 mg samples were combined with 1,000 μL extraction solution (methanol:acetonitrile:water = 2:2:1, *V*/*V*/*V*, containing 4 μg mL^–1^ internal standard). The mixture was vortexed for 30 s, following which steel beads were added and the mixture was ground at 35 Hz for 4 min and then sonicated in an ice-water bath for 5 min. The grinding was repeated and the sample was sonicated for the second time, following which it was kept at −40°C for 1 h. The sample was then centrifuged at 4°C and 10,000 rpm for 15 min, and 250 μL of the supernatant was transferred into an Eppendorf tube and dried under vacuum. The sample was reconstituted with 300 μL 50% acetonitrile, vortexed for 30 s, and sonicated in an ice-water bath for 10 min. The sample was centrifuged at 4°C and 13,000 rpm for 15 min, and 75 μL of the supernatant was transferred to a vial for analysis. Samples from the three treatments (10 μL each) were mixed to form the quality control sample for the evaluation of measurement accuracy.

For liquid chromatography-tandem mass spectrometry (LC-MS/MS) analysis, the target substances were separated using an Agilent 1290 ultra-high performance liquid chromatograph (Agilent, Waldbronn, Germany) on a Waters ACQUITY UPLC BEH Amide column (2.1 mm × 100 mm × 1.7 μm). Mobile phase A was an aqueous solution containing 25 mmol L^–1^ ammonium acetate and 25 mmol L^–1^ ammonia, and phase B was acetonitrile. Gradient elution was performed as follows: 0–0.5 min, 95% B; 0.5–7 min, 95–65% B; 7–8 min, 65–40% B; 8–9 min, 40% B; 9–9.1 min, 40–95% B; 9.1–12 min, 95% B. The mobile phase flow rate was 0.5 mL min^–1^, the oven temperature was 25°C, the sample tray temperature was 4°C, and the injection volume was 2 mL for both positive and negative ionization modes. Electrospray ionization in positive and negative modes was used for liquid chromatography-quadrupole time-of-flight (TOF) mass spectrometry to improve metabolite coverage and detection efficiency. A Triple TOF 6600 high-resolution mass spectrometer was used for data collection in information-dependent acquisition mode. In this mode, data acquisition software Analyst TF 1.7 (AB Sciex) automatically selects ions from MS for MS/MS based on pre-defined criteria. Twelve ions with high intensity (> 100 counts) were selected in each cycle for MS/MS scans, with an energy of 30 eV and a cycle time of 0.56 s. The ion source parameters were as follows: gas 1 pressure, 60 psi; gas 2 pressure, 30 psi; curtain gas pressure, 35 psi; source temperature, 600°C; declustering potential, 60 V; and ion spray voltage floating, 5,000 V (positive mode) and −4,000 V (negative mode).

#### Data Analyses

Data were processed using Excel 2016 (Microsoft Corp., Redmond, United States), One-way ANOVA was conducted using SPSS 17.0 (SPSS Inc., Chicago, United States), and Tukey’s test was used to assess the significance of differences. Metabolomic data from the mass spectra were converted to mzXML format using ProteoWizard software. XCMS was used for retention time correction as well as peak identification, extraction, integration, and alignment. The minfrac was set to 0.5 with a cutoff of 0.6. The peaks were identified using a self-developed R package and MS/MS database. In the orthogonal partial least squares-discriminant analysis (OPLS-DA) model, VIP > 1 (variable importance in the projection) of the first principal component was used in combination with *P* < 0.05 to identify metabolites that were increased or decreased in abundance. The R package software (Version 4.0.3) was used for principal component analysis (PCA) of the ionome and metabolomics. The Kyoto Encyclopedia of Genes and Genomes (KEGG) database was used in this study, and metabolic pathways were analyzed using the online MetaboAnalyst 5.0 software^1^.

## Results

### Growth Status of Cotton Seedlings Under Salt and Alkali Stress

Salt and alkali stresses significantly reduced the cotton biomass (Table 2). Compared with CK, the biomass of the leaves, stems, and roots and the total biomass of CS decreased by 47.2, 66.0, 33.0, and 51.8%, respectively. These values of AS were reduced by 58.6, 58.4, 34.6, and 53.0%, respectively.

**TABLE 2:** Dry matter weight of cotton under salt and alkali stresses.

Treatment	Biomass (g/plant)			
	Leaf	Stem	Root	Total
CK	1.45 ± 0.013 a	1.72 ± 0.009 a	0.94 ± 0.006 a	4.11 ± 0.021 a
CS	0.77 ± 0.005 b	0.59 ± 0.010 c	0.63 ± 0.003 b	1.98 ± 0.006 b
AS	0.60 ± 0.007 c	0.72 ± 0.014 b	0.62 ± 0.013 b	1.93 ± 0.017 b

*Mean ± SE.*

*Different lowercase letters after the data in the same column indicate significant differences among different treatments (P < 0.05).*

*CK, control treatment without salt or alkali stress; CS, NaCl stress treatment; AS, Na_2_CO_3_ + NaHCO_3_ stress treatment.*

### Ionomes of Cotton Seedlings Under Salt and Alkali Stress

**FIGURE 1:**
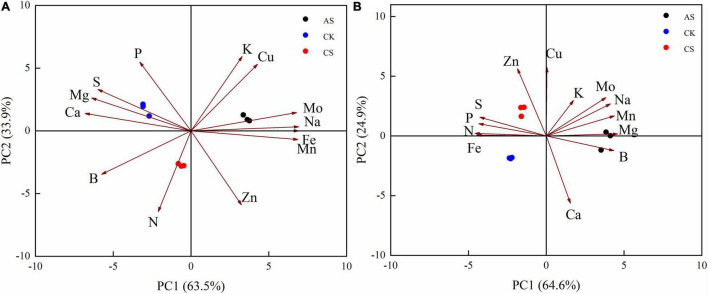
Principal component analysis of cotton. **(A)** Leaves, **(B)** Roots. CK, control treatment without salt or alkali stress; CS, NaCl stress treatment; AS, Na_2_CO_3_ + NaHCO_3_ stress treatment.

In the PCA of the Ionomes data, the total coefficients of variation of the first and second principal components for the leaves were 63.5 and 33.9%, respectively, CS and AS were well separated from CK (Figure 1). The first principal component was primarily composed of Na, Fe, Mn, Mo, Mg, and Ca, and the second principal component was composed mostly of K, N, Zn, Cu, and P (Figure 1A). The total coefficients of variation of the first and second principal components for the roots were 64.6 and 24.9%, respectively. The primary variables were N, Mg, Fe, Mn, B, and P as well as Cu, Zn, Ca, Mo, and K, respectively (Figure 1B).

**FIGURE 2:**
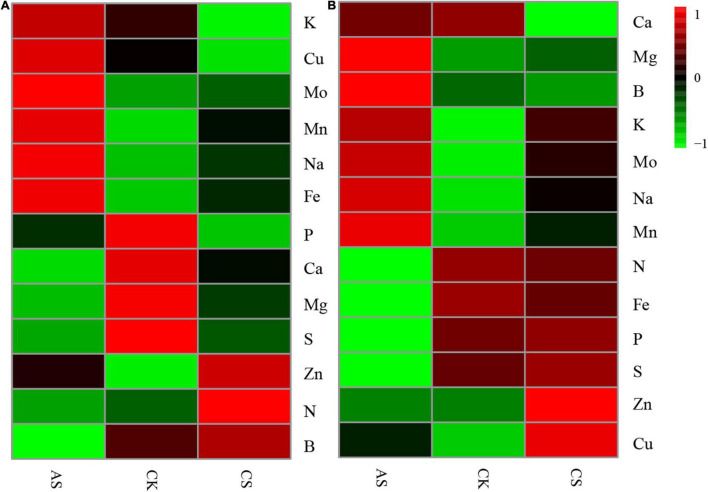
Effects of salt stress and alkali stress on ion concentration in roots and leaves of cotton. **(A)** Leaves, **(B)** Roots. CK, control treatment without salt or alkali stress; CS, NaCl stress treatment; AS, Na_2_CO_3_ + NaHCO_3_ stress treatment.

The effects of salt alkali stress on ion content in cotton organs were significantly different (Figure 2) (see Supplementary Table 1 for ion content). In the leaves (Figure 2A), CS showed increased B, N, Zn, Mn, Fe, Na, and Mo but decreased S, Mg, Ca, P, Cu, and K as compared with CK; AS showed increased K, Cu, Mo, Mn, Na, Fe, and Zn but decreased P, Ca, Mg, S, N, and B. In the roots (Figure 2B), CS showed increased Mg, K, Mo, Na, Mn, P, S, Zn, and Cu but decreased Ca, B, N, and Fe; AS showed increased Mg, B, K, Mo, Na, Mn, and Cu but decreased N, Fe, P, S, Ca, and Zn.

**FIGURE 3:**
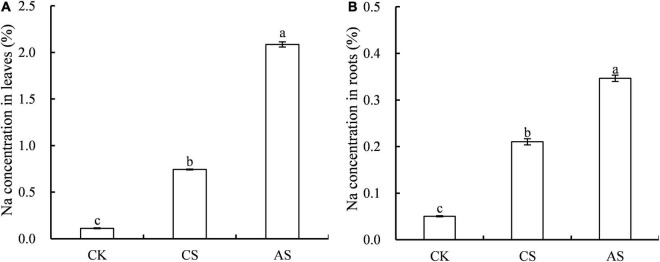
Effects of salt stress and alkali stress on Na concentrations. **(A)** Leaves, **(B)** Roots. Columns and error bars represent the mean ± standard error (*n* = 3), respectively. CK, control treatment without salt or alkali stress; CS, NaCl stress treatment; AS, Na_2_CO_3_ + NaHCO_3_ stress treatment. Different lowercases indicate significant differences among different treatments (*P* < 0.05).

The effects of salt and alkali stress on Na^+^ concentration in cotton roots and leaves were significantly different (Figure 3). Compared with CK, CS and AS showed significantly higher Na^+^, and the concentration was 5.63- and 17.6-fold higher in the leaves and 3.17- and 5.88-fold higher in the roots, respectively. The concentration of Na^+^ in AS was 1.65-fold that in CS.

### Metabolites of Cotton Seedlings Under Salt and Alkali Stress

**FIGURE 4:**
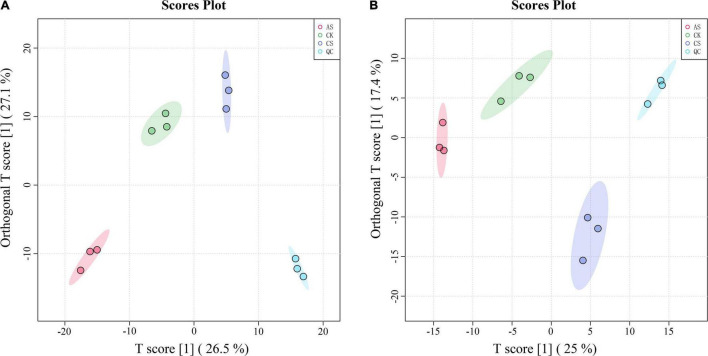
Principal component analysis (PCA) score chart of metabolites. **(A)** Leaves **(B)** Roots. CK, control treatment without salt or alkali stress; CS, NaCl stress treatment; AS, Na_2_CO_3_ + NaHCO_3_ stress treatment.

In the PCA of the metabolomic data, the first two principal components explained 53.6 and 42.4% of the variance in the leaves and roots, respectively (Figure 4). In the leaves, the primary metabolites of the first principal component included arachidonic acid, ascorbic acid 6-palmitate, pyruvaldehyde, dihydroxyacetone, and dioscin, and the primary metabolites of the second principal component included L-nicotine, indoleacetic acid, succinate, terpineol, and D-ribose 5-phosphate. In the roots, the primary metabolites of the first principal component were Glu-Lys, 3-phenylpropanoic acid, 4-hydroxybutanoic acid lactone, 3,3-dimethylacrylic acid, and 3-hydroxy-4-methoxycinnamic acid, and the primary metabolites of the second principal component were 2-amino-2-methyl-1,3- propanediol, ornithine, N-(omega)-hydroxyarginine, tyramine, and estriol 16alpha-(beta-D-glucuronide).

**FIGURE 5:**
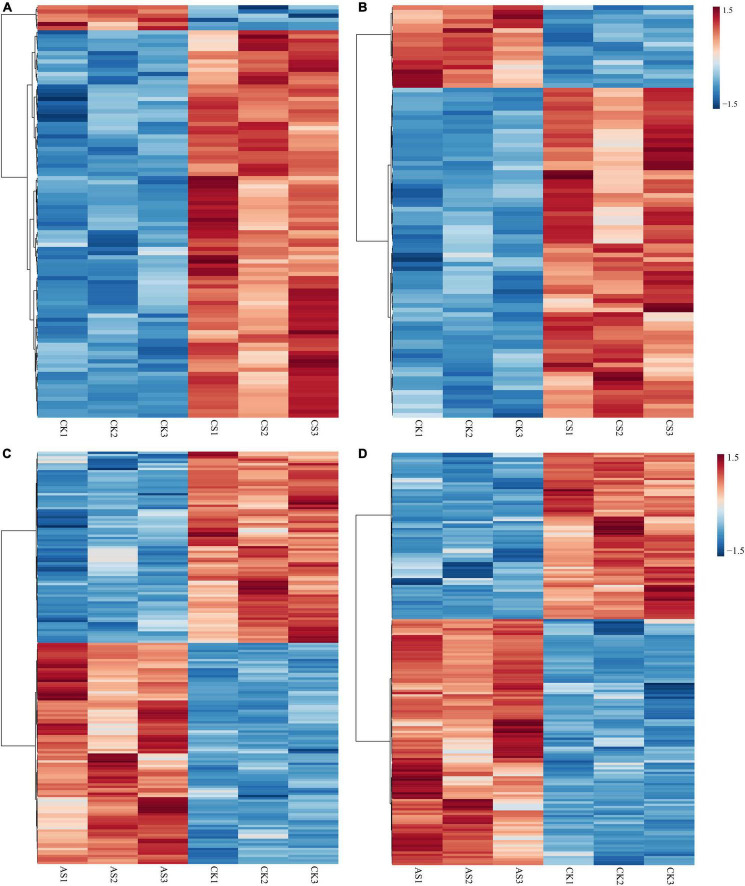
Thermographic analysis of differential metabolites in cotton roots and leaves under salt alkali stress. **(A)** CS leaves, **(B)** CS roots, **(C)** AS leaves and **(D)** AS roots. CK, control treatment without salt or alkali stress; CS, NaCl stress treatment; AS, Na_2_CO_3_ + NaHCO_3_ stress treatment.

Next, we screened the changes of differential metabolites in cotton roots and leaves under saline alkali stress (Figure 5 and Supplementary Table 2). A total of 99 DAMs were identified in CS leaves, of which 93 increased in abundance and six decreased in abundance. A total of 90 DAMs were detected in CS roots, of which 72 increased in abundance and 18 decreased in abundance. Geranylgeraniol and phosphorylcholine decreased in the leaves but increased in the roots. In AS, a total of 179 DAMs were identified in the leaves, of which 96 increased and 83 decreased in abundance; a total of 181 DAMs were identified in the roots, 108 of which increased and 73 decreased in abundance. Metabolites that decreased in the leaves but increased in the roots included pectin (galacturonic acid), succinate, 2-oxoadipic acid, *N*-acetylneuraminic acid, 3-phenylpropanoic acid, alpha-*N*-acetyl-L-glutamine, kynurenic acid, and 2-amino-2-methyl-1,3-propanediol.

**FIGURE 6:**
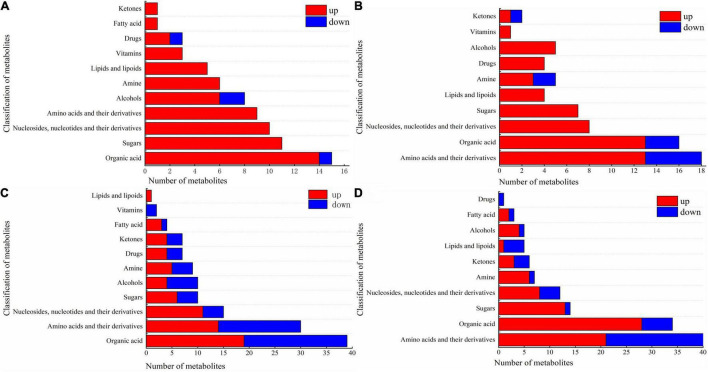
Component diagram of differential metabolites in roots and leaves of cotton under salt and alkali stress. **(A)** CS leaves, **(B)** CS roots, **(C)** AS leaves and **(D)** AS roots. CK, control treatment without salt or alkali stress; CS, NaCl stress treatment; AS, Na2CO3 + NaHCO3 stress treatment.

The various types of DAMs in the cotton leaves and roots under salt and alkali stresses are shown in Figure 6 and Supplementary Table 3. In CS leaves, one organic acid (beta-homoproline), two alcohols (geranylgeraniol and dihydrotachysterol), and one drug (phosphorylcholine) decreased, while the other metabolites increased. Cs leaves shown in Figure 6A, the increased metabolites included 14 organic acids (e.g., barbituric acid and chlorogenic acid); 11 sugars (e.g., D-mannose and sucrose); 10 nucleosides, nucleotides, and their derivatives (e.g., flavin adenine dinucleotide and 5′-deoxyadenosine); 9 amino acids and their derivatives (e.g., L-histidinol and L-tyrosine); 6 alcohols (e.g., perillyl alcohol and ribitol), and other metabolites including 6 amines, 5 lipids and lipoids, 3 vitamins, 2 drugs, 1 fatty acid, and 1 ketone. In CS roots, we also observed that a small proportion of metabolites decreased in abundance, including 5 amino acids (e.g., L-leucine and L-isoleucine), 3 organic acids (e.g., 1-aminocyclopropanecarboxylic acid and beta-homoproline), 2 amines (2-methylbutyroylcarnitine and L-carnitine), and 1 ketone (daidzein). As shown in Figure 6B, the other metabolites increased, including 13 amino acids and their derivatives (e.g., D-aspartic acid and DL-homoserine); 13 organic acids (e.g., homovanillic acid and rosolic acid); 8 nucleosides, nucleotides, and their derivatives (e.g., 5,2′-*O*-dimethyluridine and 5-methylcytidine); 7 sugars (e.g., D-lactose and sucrose); 5 alcohols (e.g., myo-inositol and geranylgeraniol); and other metabolites including 4 lipids and lipoids, 4 drugs, 3 amines, 1 vitamin, and 1 ketone.

Compared with CS, AS showed a higher diversity in metabolites, and the changes in metabolites in AS leaves are illustrated in Figure 6C. Nineteen organic acids wee increased (e.g., quinate and homocitrate) and 20 decreased in abundance (e.g., succinate and butanoic acid). Fourteen amino acids and their derivatives increased (e.g., L-arginine and L-aspartate) and 16 decreased (e.g., L-glutamine and acetyl-DL-valine). For nucleosides, nucleotides, and their derivatives, 11 increased (e.g., 5-hydroxymethylcytidine and 2′-*O*-methyluridine) and 4 decreased (e.g., 3′,5′-cyclic guanosine monophosphate and flavin mononucleotide). 6 sugars increased (e.g., arbutin and alpha-trehalose) and 4 decreased (D-quinovose and 6-phospho-D-gluconate). 4 alcohols increased (e.g., geranylgeraniol and sinapyl alcohol) and 6 decreased (e.g., coniferol and D-sorbitol). Other metabolites also observed varying degrees of increased and decreased, but the number was small (e.g., amines, drugs, ketones, fatty acids, vitamins, and lipids and lipoids). The changes in metabolites in AS roots are shown in Figure 6D. 21 amino acids and their derivatives increased (e.g., L-alanine and L-citrulline) and 19 decreased (e.g., L-isoleucine and L-threonine). 28 organic acids increased (e.g., succinate and 2-oxoadipic acid) and 6 decreased (e.g., oleanolic acid and caffeic acid). 13 sugars increased (e.g., D-fructose and glycogen) and only D-ribose 5-phosphate decreased. Other metabolites also observed varying degrees of increased and decreased, but the number was small (e.g., nucleosides, nucleotides, and their derivatives, amines, ketones, lipids and lipoids, alcohols, fatty acids, and drugs) amines, drugs, ketones, fatty acids, vitamins, and lipids and lipoids).

### Metabolic Pathways of Cotton Seedlings Under Salt and Alkali Stress

**FIGURE 7:**
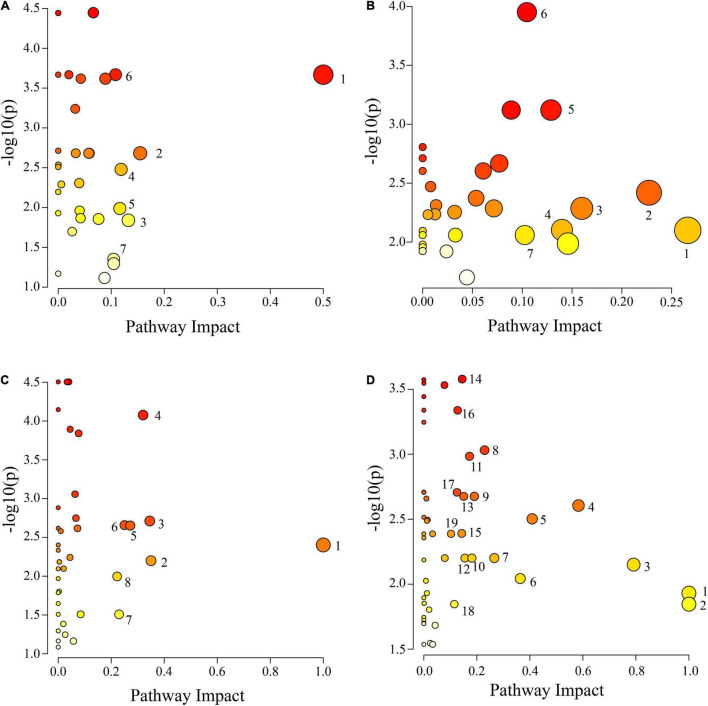
Pathway analysis of cotton leaves and roots under salt alkali stress. **(A)** CS leaves, **(B)** AS leaves, **(C)** CS roots and **(D)** AS roots. CK, control treatment without salt or alkali stress; CS, NaCl stress treatment; AS, Na_2_CO_3_ + NaHCO_3_ stress treatment. Each circle represents a metabolic pathway, the color indicates the size of the difference. The darker the color mean the more significant the difference. The lighter the color mean the less significant the difference. The size of the circle indicates the influence value. The larger the circle mean the greater the influence value, the smaller the circle, and the smaller the influence value. **(A)** 1 Isoquinoline alkaloid biosynthesis, 2 Pyruvate metabolism, 3 Stilbenoid, diarylheptanoid and gingerol biosynthesis, 4 Cyanoamino acid metabolism, 5 Pentose phosphate pathway, 6 Tyrosine metabolism and 7 Terpenoid backbone biosynthesis, **(B)** 1 Alanine, aspartate and glutamate metabolism, 2 Butanoate metabolism, 3 Pantothenate and CoA biosynthesis, 4 Arginine biosynthesis, 5 Galactose metabolism, 6 Arginine and proline metabolism and 7 Inositol phosphate metabolism. **(C)** 1 Linoleic acid metabolism, 2 Flavone and flavonol biosynthesis, 3 Pentose phosphate pathway, 4 Alanine, aspartate and glutamate metabolism, 5 Thiamine metabolism, 6 Lysine degradation, 7 Arginine and proline metabolism and 8 Tryptophan metabolism. **(D)** 1 Biosynthesis of secondary metabolites—unclassified, 2 Isoquinoline alkaloid biosynthesis, 3 Alanine, aspartate and glutamate metabolism, 4 Vitamin B6 metabolism, 5 Arginine biosynthesis, 6 Butanoate metabolism, 7 Glyoxylate and dicarboxylate metabolism, 8 Pentose phosphate pathway, 9 Starch and sucrose metabolism, 10 Citrate cycle (TCA cycle), 11 Glycerophospholipid metabolism, 12 Pyruvate metabolism, 13 Galactose metabolism, 14 Glutathione metabolism, 15 Glycine, serine and threonine metabolism, 16 Pantothenate and CoA biosynthesis, 17 Cutin, suberine and wax biosynthesis, 18 Tyrosine metabolism, 19 Inositol phosphate metabolism.

The IDs of the DAMs were imported into the KEGG database for enrichment analysis. Differential metabolic pathways were identified based on *P* ≤ 0.05, impact ≥ 0.1, and −log10 (p) value (Supplementary Table 4). The results are plotted in Figure 7. There were 7 differential metabolic pathways in CS leaves, including 4 amino acid and organic acid metabolic pathways (pyruvate metabolism, cyanoamino acid metabolism, pentose phosphate pathway, and tyrosine metabolism), and 3 other metabolic pathways (isoquinoline alkaloid biosynthesis, stilbenoid, diarylheptanoid and gingerol biosynthesis, and terpenoid backbone biosynthesis) (Figure 7A). There were 7 differential metabolic pathways in CS roots, including 3 amino acid metabolic pathways (alanine, aspartate, and glutamate metabolism; arginine biosynthesis; and arginine and proline metabolism), 3 organic acid metabolic pathways (butanoate metabolism, pantothenate and CoA biosynthesis, and inositol phosphate metabolism), and 1 sugar metabolic pathway (galactose metabolism) (Figure 7B). There were 8 differential metabolic pathways in AS leaves, including 4 amino acid metabolic pathways (alanine, aspartate and glutamate metabolism; lysine degradation; arginine and proline metabolism; and tryptophan metabolism), and 4 other metabolic pathways (linoleic acid metabolism, flavone and flavonol biosynthesis, pentose phosphate pathway, and thiamine metabolism) (Figure 7C). There were a total of 19 differential metabolic pathways in AS roots, including 7 organic acid metabolic pathways (butanoate metabolism, glyoxylate and dicarboxylate metabolism, pentose phosphate pathway, TCA cycle, pyruvate metabolism, pantothenate and CoA biosynthesis, and inositol phosphate metabolism), 5 amino acid metabolic pathways (alanine, aspartate and glutamate metabolism; arginine biosynthesis; glutathione metabolism; glycine, serine and threonine metabolism; and tyrosine metabolism), and 2 sugar metabolic pathways (starch and sucrose metabolism; galactose metabolism) (Figure 7D).

## Discussion

As a major abiotic stress limiting agricultural production, salt or alkali stress severely disrupts plant physiology, biochemistry, metabolism, and development, and inhibits plant growth (Abbas et al., 2015). In this study, salt and alkali stresses significantly inhibited cotton growth, probably as a result of a high concentration of Na^+^ leads to osmotic imbalance, membrane dysfunction, increased production of ROS, as well as the disruption of plant nutrient metabolism by high pH under alkali stress, and thus affects cell division and growth (Bracci et al., 2008; Lokhande et al., 2011; Yang and Guo, 2018). However, salt stress and alkali stress differ in their inhibition of cotton stems and leaves. Salt stress tends to inhibit stem growth, while alkali stress mostly inhibits leaf growth in this study. By analyzing the Na^+^ concentration in cotton roots and leaves under salt and alkali stresses, we found that Na^+^ under alkali stress was higher than that under salt stress. This is unexpected based on the experimental conditions. The salt content under salt stress was more than twice that under alkali stress, but the Na^+^ concentration in the cotton was higher under alkali stress. This result suggests that different osmoregulators might be involved in the response of cotton to salt and alkali stresses, which may be caused by the different nature of the stresses. The most significant difference was the oxidative stress caused by high pH under alkali stress.

Salt and alkali stresses not only inhibit nutrient uptake but also affect the transport of some elements. Our analysis of the distribution of various elements in the cotton roots and leaves indicated that salt stress inhibited the uptake of Ca, B, N, and Fe and the transport of Mg, K, P, S, and Cu, and promoted the uptake of Mo, Mn, Zn, Mg, K, P, S, and Cu and the transport of Mo, Mn, Zn, B, N, and Fe. Alkali stress inhibited the uptake of P, Ca, S, N, Fe, and Zn and the transport of Mg and B, and promoted the uptake of K, Mn, Cu, Mo, Mg, and B and the transport of K, Mn, Cu, Mo, Fe, and Zn. To adapt to salt and alkali stresses, cotton accumulated a large amount of Na^+^, and constrained Na^+^ within the vacuole through ion compartmentation. This not only promotes water absorption by cells and avoids the toxic effects of salt ions on the enzymes in the cell membrane and cytoplasm, but also helps to maintain osmotic homeostasis and ensures physiological functioning in plants (Graus et al., 2018). However, Ca^2+^ is antagonistic to Na^+^, and the excessive uptake of Na^+^ leads to Ca^2+^ deficiency in cotton. Some scholars believe that the decrease of Ca^2+^ and Fe^2+^ in roots is related to the accumulation of organic acids in roots, the organic acids formed insoluble complexes with Fe^2+^ and Ca^2+^ in the roots which impaired nutrient uptake (Sagervanshi et al., 2021). Our studies have shown that salt and alkali stresses limit the uptake of Fe^2+^ and Ca^2+^ by cotton, especially under alkali stress. Therefore, the accumulation of organic acids in roots limits the uptake of Fe^2+^ and Ca^2+^ to a certain extent. As an essential macro-element for plant growth, N uptake and transport were significantly inhibited under alkali stress. For normal N metabolism, plant roots transport NH_4_^+^ and NO_3_^–^ *via* the ammonium transporter (AMT) and nitrate transporter (NRT) protein families, respectively (Wang et al., 2012). However, high pH under alkali stress restricts the supply of external protons to the plasma membrane in the roots, which might hinder the activities of NRT and AMT, resulting in decreased uptake of NO_3_^–^ and NH_4_^+^ (Crawford and Glass, 1998).

Organic acid and amino acid metabolic pathways showed the most significant changes in the leaves under salt stress, and most metabolites were increased in abundance, including amino acids, organic acids, sugars, and alcohols. The same trend was observed in the roots, with significant accumulation of amino acids, organic acids, sugars, and alcohols. There are reports that plants accumulate compatible solutes (low-molecular-weight metabolites) such as amino acids, carbohydrates and polyphenols, and organic acids (Liang et al., 2022). Salt and alkali stresses induce osmotic stress. To adapt to osmotic balance between their cytoplasm and environment, plants accumulate large amounts of small-molecule organic solutes. When plants are subjected to abiotic stress, amino acids serve as important compatible solutes, of which proline is the most well-known and plays an important role in osmoregulation and cell membrane stability (Widodo et al., 2009), thus promoting the growth of salt-tolerant plants under salt stress (Wu et al., 2013b; Yang et al., 2017). As the intermediates or final products of some metabolic pathways, amino acids participate in the regulation of various biochemical pathways, play prominent functions in protein biosynthesis and participate in signaling processes during plant stress response, thereby affecting many physiological processes in plants (Amir et al., 2018; Yang et al., 2018; Wang et al., 2020). Tyrosine is not only a central hub of many metabolic pathways, but also the precursor of special metabolites such as non-protein amino acids, attractants, and defense compounds (Schenck and Maeda, 2018), playing an important role in plant adaptation to stress (Datta et al., 2016). Moreover, amino acids that accumulate under various abiotic stresses can act as effective antioxidants to scavenge free radicals in plant cells (Caldana et al., 2011). Studies have shown that sucrose is a compatible osmoprotectant in osmoregulation and detoxification (Singh et al., 2015; Yang et al., 2018). Sugars not only alleviate osmotic stress but also serve as important energy sources. Yang et al. (2009) reported that glucose, fructose, and sucrose increased under neutral salt stress. Sugars are energy sources and structural components of cells, and sucrose is the primary carbon source and energy source for plant metabolism (Fougère et al., 1991; Kaplan et al., 2004; Rosa et al., 2009). Plants provide energy for osmotic homeostasis in cells through the decomposition of polysaccharides stored as a carbon source.

**FIGURE 8:**
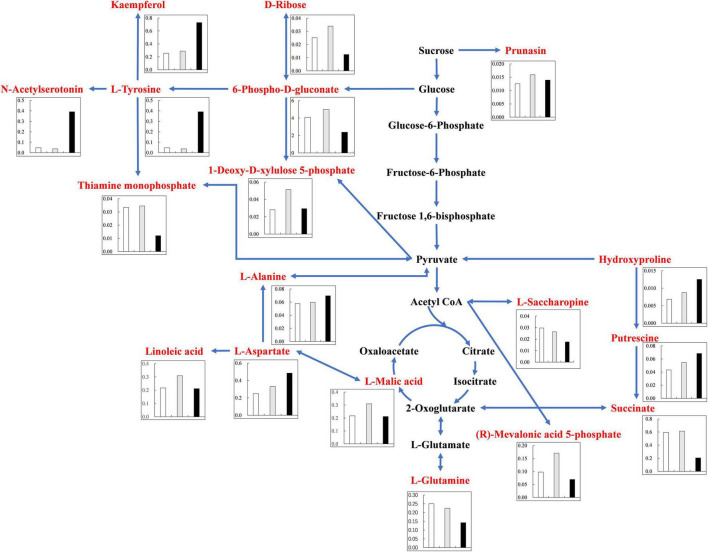
Changes of KEGG pathway metabolites in Cotton Leaves under salt alkali stress. The histogram describes the content of corresponding differential metabolites under the three treatments. The control was white, gray under salt stress and black under alkali stress. Metabolic pathways were constructed according to KEGG (http://www.genome.jp/kegg/) metabolic database.

**FIGURE 9:**
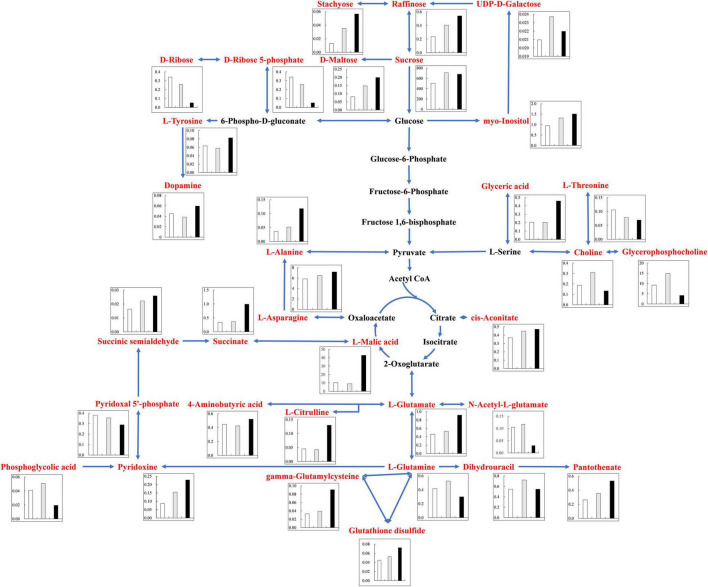
Changes of KEGG pathway metabolites in Cotton Roots under salt alkali stress. The histogram describes the content of corresponding differential metabolites under the three treatments. The control was white, gray under salt stress and black under alkali stress. Metabolic pathways were constructed according to KEGG (http://www.genome.jp/kegg/) metabolic database.

**FIGURE 10:**
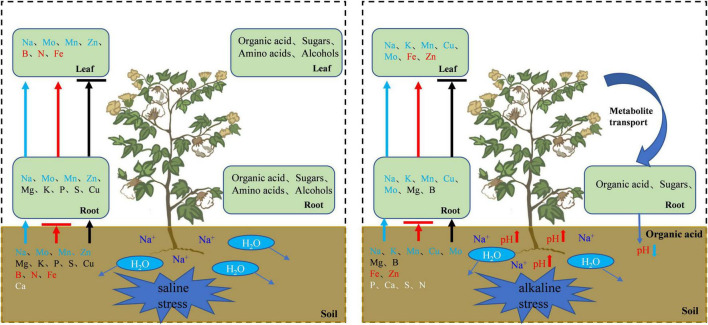
Schematic diagram of cotton regulation mechanism under salt alkali stress. The arrow indicates the transport direction of ions of the same color, and the horizontal line indicates that the transport is blocked. White letters indicate that plant absorption is inhibited.

Under alkali stress, linoleic acid metabolism showed the greatest changes in the leaves, and β-oxidation is the primary means of fatty acid decomposition, which provides large amounts of energy for life activities and is critical for the plant stress response (Xu et al., 2008). Under alkali stress, palmitic acid and linoleic acid accumulated in the leaves in large quantities, which might promote β-oxidation to produce energy. Zhang et al. (2016) also found that the accumulation of large amounts of fatty acids in leaves would promote β-oxidation, generating large amounts of energy, which reduce the damage caused by a hostile environment. However, other metabolites in leaves did not accumulate significantly, but increased by half and decreased by half. This indicates that the organic solutes in the leaves for osmoregulation may be replaced by inorganic ions while their metabolites perform other physiological functions. The KEGG pathway analysis revealed that metabolites associated with organic acid metabolism increased, including organic acids as well as sugars and amino acids that can be converted to organic acids (Figures 8, 9). Organic acid metabolism serves a key role in the plant response to alkali stress. Under alkali stress, Na^+^ influx dramatically increases the positive charge in plant cells, and a significant reduction of anions (Cl^–^, NO_3_^–^, H_2_PO_4_^–^) causes a severe deficit in negative charge. The secretion of organic acids in the root system can compensate for the negative charge caused by inorganic ions and maintain intracellular pH stability (Pang et al., 2016). Sugars, such as raffinose and sucrose, are located at the most upstream of metabolic pathways. Both these sugars significantly increased in the cotton roots under salt and alkali stresses, of which raffinose was significantly higher under alkali stress than under salt stress. Downstream of these two metabolites are amino acid and organic acid metabolism. It is well-known that 30–40% of photosynthetically fixed carbon is allocated to the rhizosphere as root exudates (Badri and Vivanco, 2009). The increased sugars in the roots suggests that more photosynthates were transported from the leaves to the roots, and we speculate that cotton under alkali stress transports more photosynthates to the roots for conversion to organic acids rather than for increasing leaf biomass. Aspartate, asparagine, arginine, glutamate, ornithine, threonine, and homoserine also significantly accumulated under alkali stress. Similarly, Ahmad et al. (2021) found that ornithine, aspartate, asparagine accumulated significantly under alkali stress. ornithine occupies a vital role in the arginine pathways. Ornithine together with arginine, can be converted to polyamines that participate in the plant response to stress (Anwar et al., 2018; Yang et al., 2020). Aspartate is important for the biosynthesis of asparagine, as well as the circulation, storage, and transportation of N in various organs (Gaufichon et al., 2016). The relationship between aspartate and asparagine is closely associated with the catabolism of many metabolites, and the conversion of aspartate to asparagine is essential for the supply of N (Han et al., 2021). Moreover, asparaginase catalyzes the conversion of asparagine to aspartate, which can be used for amino acid biosynthesis (Gaufichon et al., 2016). Homoserine and threonine are also derived from the aspartate pathway, and both are important for improving plant stress tolerance (Zeier, 2013). Aspartate and glutamate can link amino acid metabolism to organic acids in the TCA cycle and to carbon metabolism in glycolysis (Kirma et al., 2012). Similarly, it has been reported that under alkali and salt stress, the TCA cycle of plants increases, releases more energy and accelerates the physiological and metabolic response to stress (Guo et al., 2017). Central metabolism can be fine-tuned through glycolysis (e.g., sucrose, hexose, and pyruvate) and the TCA cycle (e.g., 2-oxoglutarate and succinate) to support cell survival (Sullivan et al., 2015; Jia et al., 2016; Ullah et al., 2017). Under alkali stress, cotton is induced to secrete large amounts of organic acids, which can act as a buffer to help plants resist environmental changes and maintain intracellular pH stability and ion homeostasis (Yang et al., 2010; He et al., 2011; Yang, 2012). The present study showed that succinic acid, malic acid, and *cis*-aconitate, which are important intermediates in the TCA cycle, were increased in the cotton roots under alkali stress. The organic acids accumulated in the TCA cycle and downstream metabolism can neutralize excessive cations in the cells and regulate intracellular pH (Yang et al., 2007). The TCA cycle also serves as an important energy source in cotton under alkali stress. In the leaves, however, succinic acid, malic acid, and *cis*-aconitate were significantly decreased in abundance. These results suggest that cotton roots play a key role in tolerance to alkali conditions under salt stress, and that organic acid metabolism in the roots is the key process involved in this tolerance. The mapping of our results may help to clarify the salt and alkali tolerance mechanism of cotton (Figure 10).

## Conclusion

Under salt stress, the uptake of P, Mg, K, S, and Cu increased but their transport decreased, while the uptake and transport of Mn, Zn, and Mo were improved, and the transport of B, N, and Fe was increased. Small-molecule soluble substances such as organic acids, sugars, and amino acids accumulated significantly in cotton, which have a primary role in osmoregulation. The metabolism of amino acids and organic acids was important for enhancing salt tolerance, while sugar metabolism was mainly used as a source of energy. Under alkali stress, the uptake and transport of N, P, S, and Ca were inhibited, whereas the uptake and transport of Mn, Mo, Cu, and K were improved, the uptake of Mg and B was promoted but their transport was inhibited, and the transport of Zn and Fe was increased. β-Oxidation, TCA cycle, and glycolysis were enhanced to provide the energy needed for life activities. Sugars and other organic substances produced by leaves are transported to roots and transformed into organic acids, and the accumulation and metabolism of organic acids in roots are promoted, thereby helping to maintain intracellular ion homeostasis and counteract the negative effect of high pH under alkali stress. Therefore, foliar spraying of nutrient elements such as N, P, K, CA, Mg, S, B, Fe, Cu and Ni and organic fertilizers such as organic acid, sugar and amino acid under salt stress, foliar spraying of nutrient elements such as N, P, CA, Mg, S, Fe, Zn and B under alkali stress and soil application of organic fertilizer rich in organic acid may alleviate the adverse effects of salt stress on cotton.

## Data Availability Statement

The original contributions presented in the study are included in the article/Supplementary Material, further inquiries can be directed to the corresponding author/s.

## Author Contributions

JG and WM conceived of this study, participated in the design of this study, and helped to draft the manuscript. JG and HG performed the statistical analysis. JG carried out the study, together with XL and drafted the manuscript. YT collected the important background information. All authors read and approved the final manuscript.

## Conflict of Interest

The authors declare that the research was conducted in the absence of any commercial or financial relationships that could be construed as a potential conflict of interest.

## Publisher’s Note

All claims expressed in this article are solely those of the authors and do not necessarily represent those of their affiliated organizations, or those of the publisher, the editors and the reviewers. Any product that may be evaluated in this article, or claim that may be made by its manufacturer, is not guaranteed or endorsed by the publisher.

## Footnotes

1
https://www.metaboanalyst.ca

## Abbreviations

EC: Electrical conductivity
One-way ANOVA: One-way analysis of variance
PCA: principal component analysis
KEGG: Kyoto Encyclopedia of Genes and Genomes
psi: Pounds per square inch
DAMs: Differentially accumulated metabolites
OPLS-DA: orthogonal partial least squares-discriminant analysis
VIP: variable importance in the projection
Glu: Glutamate
Lys: Lysine
ID: Identity document
TCA cycle: tricarboxylic acid cycle
AMT: ammonium transporter
NRT: nitrate transporter.
